# Responding to the challenge of Adolescent Per*i*natal Depression (RAP*i*D): protocol for a cluster randomized hybrid trial of psychosocial intervention in primary maternal care

**DOI:** 10.1186/s13063-020-4086-9

**Published:** 2020-02-27

**Authors:** Oye Gureje, Lola Kola, Bibilola D. Oladeji, Jibril Abdulmalik, Olatunde Ayinde, Phyllis Zelkowitz, Ian Bennett

**Affiliations:** 1 WHO Collaborating Centre for Research and Training in Mental Health, Neurosciences and Substance Abuse, Department of Psychiatry, University of Ibadan, Ibadan, Nigeria; 2 Department of Psychiatry, University of Ibadan, Ibadan, Nigeria; 3 Division of Social and Transcultural Psychiatry, McGill University, Montreal, Canada; 4 Department of Psychiatry, Jewish General Hospital, Montreal, Canada; 5 Departments of Family Medicine, Psychiatry and Behavioural Science, and Global Health, University of Washington, Seattle, WA USA

**Keywords:** Adolescent, Perinatal depression, Effectiveness–implementation research, Nigeria

## Abstract

**Background:**

Adolescent pregnancy is a pressing public health issue globally, and particularly in low and middle-income countries. Depression occurring in the perinatal period is common among women and more so among adolescent mothers. Effective treatments for the condition have been demonstrated in adults but the needs of adolescents are often unique, making such treatments unlikely to meet those needs.

**Method/study design:**

A hybrid effectiveness–implementation research study is described in which a cluster randomized trial design is used to explore the effectiveness as well as the utility in routine practice of an intervention package specifically designed for adolescents with perinatal depression. Consenting pregnant adolescents (aged less than 20 years) who are newly registered for antenatal care are enrolled into the trial if their fetal gestational age is less than 36 weeks and they score 12 or more on the Edinburgh Postnatal Depression Scale (EPDS). The intervention package consists of structured sessions of behavior activation, problem-solving treatment, and parenting skills training, and is delivered by primary maternal health care providers, complemented by support provided by a “neighborhood mother” identified by the adolescent. Mothers in the control arm receive care as usual. The trial is conducted in clinics where the maternal providers are trained to deliver routine depression care with the use of the WHO Mental Health Gap Action Programme, intervention guide. Assessments are undertaken by trained blinded assessors at baseline, at childbirth, and at 3 and 6 months postpartum. The primary outcome, assessed at 6 months, is the level of maternal depression (measured with the EPDS). The secondary outcome is parenting skills (assessed with the Home Observation Measurement of the Environment, Infant–Toddler version), while tertiary outcomes include measures of disability, quality of life, mother–child bonding, as well as infants’ nutritional and growth indices.

**Discussion:**

This, to the best of our knowledge, will be the first fully-powered trial of an intervention package specifically designed to address the unique needs of adolescents with perinatal depression.

**Trial registration:**

ISRCTN16775958. Registered on 30 April 2019.

## Background and rationale

Globally, adolescent pregnancy is a pressing public health issue [1]. Pregnancy in adolescents complicates the biological and psychological changes taking place in their bodies and also complicates the expectations of their social roles beyond what the average pregnant adult would experience. Often associated with social determinants of health such as poverty and illiteracy [2], the problem is more pressing in low and middle-income countries (LMICs). The World Health Organization estimates that despite the declining global rates of adolescent pregnancy, up to 11% of all births worldwide are still to girls aged between 15 and 19 years [3]. The global rate for adolescent pregnancy in the 2015 World Health Statistics is put at about 44 per 1000 girls between the ages of 15 and 19 years, with a range of 1–201 across countries, with the highest rates in countries in sub-Saharan Africa [4]. In Nigeria, for example, about 31% of women have had a live birth before age 18 years [5].

Reports suggest that self-reported depression in perinatal adolescents may range between 8 and 47%, depending on the period and method of evaluation [6]. Using a clinician-administered structured interview, rates of 16% for major depressive disorder during pregnancy, 20% at 6 weeks postpartum, and 26% at 1 year postpartum have been reported [6]. Although widely variable, the reported rates of perinatal depression suggest that the problem may be higher than that commonly found among perinatal adults [7, 8].

The consequences of perinatal depression are considerable for both the mother and the infant [9]. As in adults, adolescent perinatal depression is a risk factor for preterm birth and low birth weight, in particular with depression occurring in the second and third trimesters [6]. The effects of perinatal depression on the babies in LMICs can be long lasting [10]. More than in adults, adolescent perinatal depression is associated with other unique consequences such as increased risk of further pregnancy in adolescence, the use of aggressive parenting behaviors, stunting and cognitive delays in the infants, preschool problem behavior, poorer school performance, and higher levels of psychopathology in the child at 14 years of age [6, 10]. Many of these consequences reflect poorer adjustment to motherhood and poorer parenting skills [8].

Worldwide, only a minority of depressed persons get the care they need [11]. The situation is significantly worse in LMICs [12]. The treatment gap for depressed perinatal adolescents may even be much larger given that many health systems are not attuned to their unique care needs during the period of pregnancy. The ensuing age and gender inequity is only likely to be more profound in LIMCs where the mental health service is often characterized by extreme resource scarcity and inefficiency as well as pervasive stigmatization of mental illness in the community [13].

Most previous research on perinatal depression has focused on the consequences and treatment of perinatal depression in adults. Few studies have examined the effectiveness of interventions delivered to adolescents with perinatal depression. In a systematic review of the literature conducted as recently as 2014, Lieberman et al. [14] were able to identify only two treatment studies. One study evaluated the effectiveness of group interpersonal therapy among 11 pregnant girls (mean age 16.5 years) with DSM-IV major depression over a 12-week period. The other study assessed the impact of a telephone-based depression collaborative care program consisting of motivational interviewing and psychoeducation over 6 months among 97 teenage mothers (mean age 16.4 years). Both studies reported positive outcomes in the adolescents following treatment. Neither of the studies examined the impact of treatment on the infants or used a randomized controlled design. The treatment of perinatal depression among adolescents is therefore one of the major neglected areas of public health care for adolescents worldwide.

In a recently concluded cluster randomized controlled trial of interventions for perinatal depression in primary care in Oyo State, Nigeria [15], we had a chance to do some explorations of what an appropriate intervention for adolescents with perinatal depression might consist of in order for it to meet the complex and unique needs of this group of mothers. In that trial, 772 teenage adolescents (aged ≤ 19 years; mean age 18 years) were among the entire sample of 9352 persons screened for perinatal depression in the second or third trimester (that is, 8.3% of the entire sample). Of the screened population, 727 met DSM-IV criteria for major depression. Of these, 137 were adolescents (mean age 17.8 years), representing a prevalence of 18.8% in this age group, compared to 6.9% in those aged 20 years and over. Of the 137 l,131 adolescents provided consent and were entered into the trial. While the pattern of recovery from perinatal depression by the participating adolescents was similar to that of adults, the adolescents nevertheless performed worse than adults at the 6-month outcome on indices of parenting skills. Specifically, adolescent mothers were rated significantly lower on overall scores on the Home Inventory for Measurement of the Environment, Infant–Toddler version (HOME-IT) [16], as well as on the subscale scores of responsivity and involvement [8].

The findings of that study suggest that, for adolescent mothers, interventions targeted at improving parenting skills should be a necessary component of effective interventions for perinatal depression. While there are hardly any studies of parenting skills intervention in adolescents with perinatal depression, there is evidence suggesting that interventions designed to provide parent education and improve parent–infant interactions for women with perinatal disorders show some promise. For example, parenting skills interventions for adolescent mothers without mental health problems may improve the social, emotional, and cognitive outcomes of their children [17].

### Objectives

The main objectives of the Responding to the challenge of Adolescent Perinatal Depression (RAP*i*D) trial are as follows: to design an intervention package that relieves the symptoms of adolescent perinatal depression and improves their parenting skills; to compare the effectiveness of the intervention with care as usual; and to explore factors that may facilitate the routine use of the intervention in primary maternal health care.

#### Primary hypotheses

1. Among adolescents presenting with depression during pregnancy, the intervention package will, at 6 months postpartum, produce significant improvement in depression symptoms as assessed by the EPDS. For the purpose of this study, a mean total EPDS score difference of 2.0 between the arms will be regarded as a clinically meaningful difference in depression symptoms (see “Sample size determination for the RCT”).

2. The second primary hypothesis is that mothers receiving the study intervention will have significantly better parenting skills at 6-month postnatal follow-up compared to those receiving usual care as assessed with the HOME-IT [18, 19].

## Method/design

This hybrid type 1 (“effectiveness – implementation”) study [20] will combine a single-blinded, randomized controlled trial to examine effectiveness with an implementation research to investigate contextual factors that affect maternal care providers' acceptability of and fidelity to the intervention and adolescent mothers’ adherence to and satisfaction with treatment. To achieve this, we will utilize a mixed-methods design, drawing on quantitative and qualitative approaches.

### Data collection procedure

#### Formative study

A series of formative activities took place at the beginning of the project. We conducted engagement meetings with key stakeholders consisting of decision-makers at the State Primary Health Care Board and various cadres of maternal frontline providers, including supervisory physicians, facility managers, community health workers (CHO), and community health extension workers (CHEW). Following the meetings, key informant interviews were held with frontline maternal care providers and with adolescent mothers. The key informants were selected from among providers and adolescent trial participants in our previously concluded RCT [15]. In the interviews with providers, we explored facility manpower and workload issues; experience with the delivery of treatment during the RCT, in particular engagement with the adolescents; and facility organizational issues, including those relating to the scheduling of clinic appointments for trial participants. With the mothers, who were adolescents during the trial, we explored perception of the appropriateness and usefulness of the services received by them, especially with regard to coping with parenting roles following childbirth; their level of satisfaction with the care they received; and what was lacking in the care received. With both groups we explored how evidence-based care for perinatal depression, including the provision of parenting skills, could be integrated into routine maternal care for adolescent mothers. Finally, we conducted a theory-of-change workshop with selected stakeholders to map the route from intervention design to delivery to expected outcome, identifying facilitators and potential barriers to successful project implementation.

### Effectiveness phase (randomized controlled trial)

#### Study setting

This is a single-blind, cluster randomized trial. The study is taking place in primary maternal care clinics (MCCs) in Oyo State, Nigeria. These clinics were selected from across the 11 local government areas (LGAs) in and around the city of Ibadan. In Oyo State, maternal and child health care services are provided in community MCCs by primary health care workers (PHCW), with the different cadres consisting of nurse/midwives, CHO, and CHEW. However, the CHO and CHEW are the main direct clinical service providers. Along with training to provide care for persons with a range of common health problems presenting in primary care, all have also received basic midwifery training. Supervision is routinely provided to the frontline providers by general physicians, one each supervising a group of clinics located in the local government area. When required, these physicians make referrals to specialists, including psychiatrists, at either one of the two institutions with mental health specialists in the city.

##### Eligibility and randomization

The unit of allocation is the primary maternal care clinics. Using the methods described by Raab and Butcher [21], the allocation of primary maternal care clinics to trial arms is balanced according to the following characteristics: LGA (rural/urban) and clinic patient population (large/low).

Eligible clinics were those offering maternal and child health services and whose staff have also received prior training in the use of the WHO Mental Health Gap Action Programme, intervention guide (mhGAP-IG) and were therefore offering at least some basic evidence-based treatments for persons with depression, including women with perinatal depression. Following description of the study to the facility managers, only clinics whose facility managers consented to participate and had a full complement of staff sufficient to allow their effective participation in the study while continuing with their routine service were randomized into the trial. Eligible and consenting clinics were stratified by local government areas and randomized to the intervention or control arm of the trial using a computer-generated number sequence by a statistician who had no other involvement with the study procedure.

##### Ethics and research governance

The study was approved by the University of Ibadan/University College Hospital Ibadan Joint Ethical Review Committee, thus ensuring that it is being conducted in accordance with international standard ethical guidelines and in compliance with the specifications of the Nigerian National Code for Health Research Ethics. An independent Trial Steering Committee (TSC) is monitoring and supervising the implementation of the trial, ensuring that the approved protocol is being strictly followed. The TSC meets face-to-face twice in a year and has teleconferences scheduled by the Chairperson based on any information from the Principal Investigator that the Chairperson considers of sufficient importance for immediate deliberation by the members of the TSC, including adverse events. Membership of the TSC consists of an adult psychiatrist, a child and adolescent psychiatrist, a social worker, a frontline maternal care provider, a trialist, and a maternal mental health service user. A Trial Management Committee (TMC) provides technical oversight and operational direction for the trial. Its members are the Principal Investigator, the co-investigators, the Trial Manager, and the Study Coordinator. The TMC has bimonthly teleconferences and annual face-to-face meetings. A Project Team consisting of the Trial manager, study coordinator, and field supervisors is responsible for the day-to-day conduct of the trial.

Important modifications to the protocol during the conduct of the trial are communicated to both the Ethical Review Committee and the TSC, and approval is sought for them.

##### Enrollment and informed consent procedure

Consecutive newly registered women presenting for antenatal care are approached while waiting in the clinic reception to see the maternal care provider and those who agree are screened with the Edinburgh Postnatal Depression Scale (EPDS) by trained research staff. Women most commonly register for antenatal care early in the second trimester in these clinics. Those who screen positive by scoring 12 or more on the EPDS are provided with the full study details and invited to be assessed further for enrollment in the trial. Those who consent to be further assessed are interviewed for inclusion and exclusion criteria and, if eligible, invited to enter the trial. Those who meet the eligibility criteria and provide signed informed consent to participate are provided with their EPDS scores, which are to be handed over to their maternal care provider.

The inclusion criteria are as follows (patients must satisfy all of the following to be considered for study entry):

- Adolescents aged less than 20 years
- Must score 12 or more on the Edinburgh Postnatal Depression Scale, a score that we have found to reliably identify persons meeting the criteria of DSM-V major depression of at least moderate intensity
- Fetal gestational age less than 36 weeks
- Provide signed informed consent (if less than 16 years of age, a parent or guardian must also provide signed consent)

The exclusion criteria are as follows (patients will be excluded from study entry if any of the following are met):

- Immediate need for medical attention
- Actively suicidal (a structured approach for identifying risk of suicide in those enrolled in the trial and for responding appropriately is being implemented)
- Unlikely to be available for follow-up at the 6-month postnatal period

### Schedule of trial recruitment and participation

**Fig. 1:**
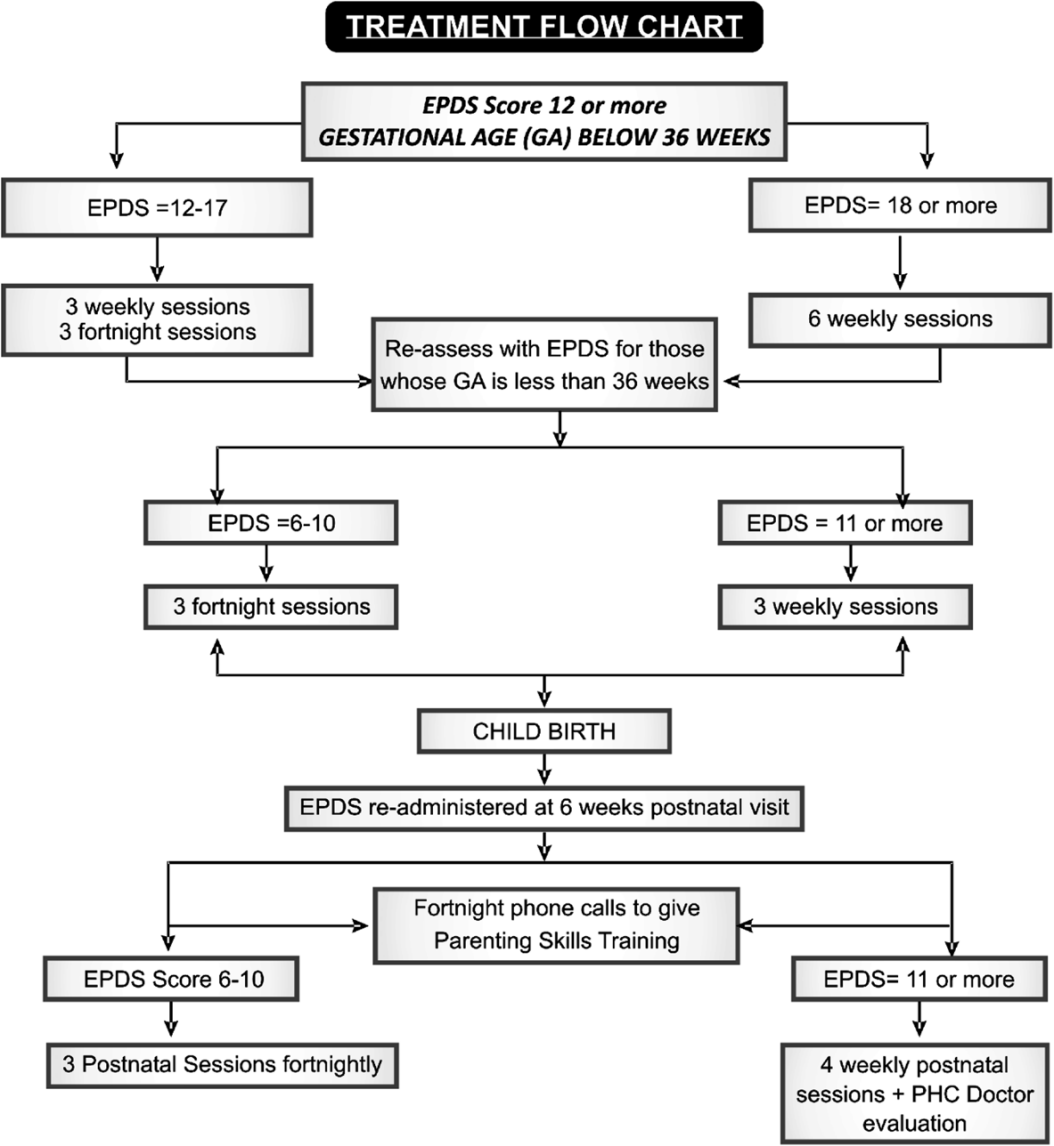
Trial profile (PHC primary health care)

Enrollment into the trial commenced on 15 May 2018 and is projected to end 15 November 2019. The last 6-month postnatal outcome assessment is expected to occur in August 2020. The schedule of enrollment and assessments follows the Standard Protocol Items: Recommendations for Interventional Trials (SPIRIT) (see Table 1 and Fig. 1).

**Table 1:** Follow-up assessment time points for mothers

	Baseline	At childbirth	3 months	6 months
Edinburgh Postnatal Depression Scale (EPDS)	X		X	X
Generalized Anxiety Disorder Assessment (GAD-7)	X		X	X
WHO Quality of Life-BREF (WHOQOL-BREF)	X		X	X
WHO Disability Assessment Scale—12 item version	X		X	X
Postnatal Bonding Questionnaire			X	X
Maternal Adjustment and Maternal Attitudes assessment questionnaire—prenatal version	X			
Maternal Adjustment and Maternal Attitudes assessment questionnaire—postnatal version			X	X
Home Inventory for Measurement of Home Environment, Infant–Toddler version (HOME-IT)				X
Delivery information (where, when, type) Baby (birth weight, height, and head circumference)		X		
Infant nutrition, immunization, disease, developmental milestones			X	X
Client satisfaction questionnaire (CSQ)				X
Family planning and new pregnancy questionnaire				X
Perinatal Infant Care Social Support scale (PICSS)				X

### Training of providers

Providers in both arms of the study are selected from facilities where the staff use the mhGAP-IG to assess and treat patients following their training in the use of the tool. For this study, and before the recruitment of the first patient, the providers in the intervention arm participated in a 3-day training workshop focused on the delivery of behavior activation, problem-solving treatment, and parenting skills training to the adolescents. Providers also received instructions on how to engage with and enlist the involvement of the “neighborhood mother” (see later). Training was conducted by psychiatrists with extensive experience in providing such training (BDO, JA, and OA) and consisted of didactic lectures delivered within an interactive approach as well as role plays. A 1-day refresher training was conducted about 3 months into the trial once recruitment and intervention had started, The refresher training provided the opportunity to review real-live experiences of the providers with patients and to use case examples to build on core competencies.

Research assistants recruited and trained for the trial all have at least a college degree and are experienced in the assessment of subjects for trials. They were given 5-day training in the administration of the study instruments and general study procedure. They conducted an interrater exercise on the HOME-IT and other tools during their training.

### Interventions

#### Intervention arm

**Fig. 2:**
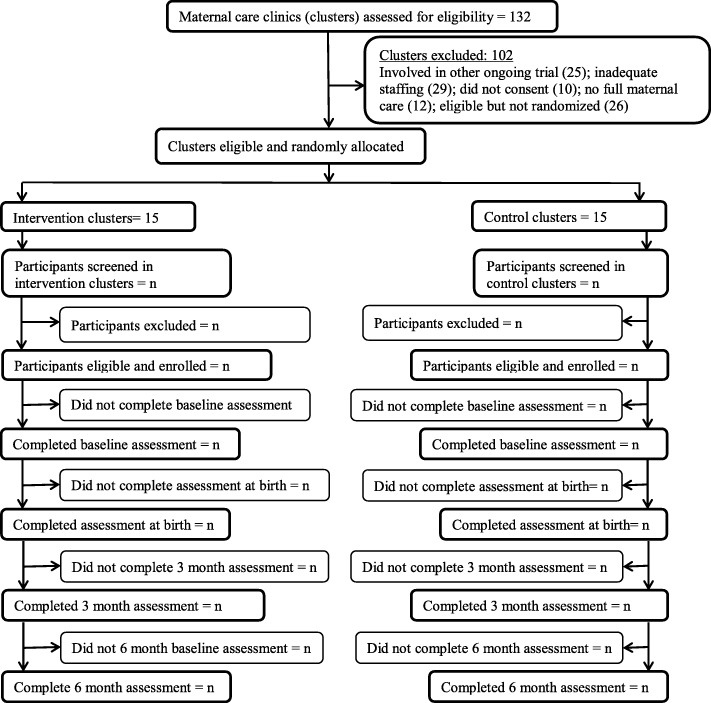
Treatment flow chart. EPDS Edinburgh Postnatal Depression Scale

Adolescents in the study intervention arm receive a manualized package of care that consists of: behavioral activation and problem-solving treatment; parenting skills training; and social and parenting skills support provided by a “neighborhood mother”. The behavioral activation and problem-solving treatment is delivered in six sessions during the antenatal period, consisting of three weekly sessions followed by three fortnightly sessions for those with EPDS scores of 12–17, or all six sessions delivered weekly for those with EPDS scores > 17. Supplemental sessions may be delivered if, during the 6-week postnatal visit, the providers find that the mother still has significant levels of depression (EPDS score of 6 or more). The number and frequency of these sessions are determined by the providers based on the level of depression (see Fig. 2). Treatment adherence is promoted by providers calling or sending text messages to their patients reminding them of appointments and agreed homework tasks from the PST sessions. This approach and the format of the problem-solving treatment are similar to what we used in our earlier RCT for perinatal depression. The parenting skills training is delivered in two ways: as a component of the problem-solving treatment which is provided in face-to-face sessions; and through mobile phone calls and texts (as appropriate) delivered in the postnatal period. There is a core set of themes to guide the provider during the calls, but attention to the particular needs or deficits of individual adolescent mothers is also addressed during the calls. The core themes consist of: personal and health care needs during pregnancy, including nutrition, rest, exercise, avoiding alcohol, and self-medication; preparing for childbirth; early signs of common ill health during pregnancy and in the infant; care of the newborn; infant feeding (including good and common bad practices to avoid); immunization schedule; stimulating and responding to infant’s needs; and dispelling cultural myths and taboos that are either harmful or unhelpful to good parenting. Many of the materials to inform the contents of the parenting skills training have been designed to address deficits in parenting skills among adolescent mothers as observed in our previous RCT. The materials also reflect information obtained during the formative qualitative activities. Providers make fortnightly calls starting shortly after the 6-week postnatal clinic visit of the mother and child, and continue until at least the 6-month postnatal outcome assessment. (We will encourage providers to continue with this, as needed, even after the discontinuation of the mother from the trial at the end of the 6-month postnatal period.)

A third component of the intervention package is the engagement of a “neighborhood mother” in the provision of social support and complementary parenting skills training to the adolescent. The idea of a “neighborhood mother” came from the extensive preliminary qualitative interviews with the adolescents when two observations were made: it is not uncommon to find that pregnant adolescents have been ostracized by their biological parents due to conflict related to the pregnancy, and may also not have support from the spouse’s parents; and as a result of this ostracization, the adolescent is often lacking in support from an experienced female who may themselves have nurtured children and can guide the adolescent in the basics of child care. For the purpose of this trial, the adolescent in the intervention arm is encouraged to identify a female in the neighborhood, who may or may not be biologically related, but who the adolescent can repose confidence in for needed social and instrumental support. The woman so identified is invited to the clinic with the adolescent for briefing by the maternal care provider and is enlisted to provide hands-on parenting skills training and support to the adolescent. The neighborhood mother agrees to work with the maternal care provider to address any identified skills deficits the adolescent may have. At each clinic visit and during postnatal telephone calls, the provider checks with the adolescent how the relationship with the neighborhood mother is going. The provider also makes regular telephone contact with the neighborhood mother to exchange experience on progress with addressing the adolescent’s needs.

#### Control arm

Participants in the control arm receive usual care. As in the intervention clinics, providers in the control clinics have had prior training in the use of the mhGAP-IG. Usual care for perinatal depression in these facilities thus consists of the basic specifications of the mhGAP-IG for treating depression, and these include psychoeducation, reactivation of the social network, and addressing current psychosocial stressors. Providers decide on the number of sessions even though, if implemented according to the guide, patients with depression are expected to be seen a number of times. In this arm, there are no structured sessions of behavior activation and problem-solving treatment, no structured parenting skills training, and no engagement of a “neighborhood mother” in the provision of care to the adolescent.

### Outcome evaluation and instruments

Outcome assessments are conducted in participants’ homes or at any other place of their preference. Outcome assessors have no involvement in the delivery of the intervention and conduct the assessment blind to the participant’s study arm. Assessments of participants are being conducted at baseline (within 72 h following enrollment) and at the 3-month and 6-month postnatal periods (Table 1). Details of childbirth are obtained from the facility where delivery takes place, from the mother, and by direct measurement of the baby soon after childbirth following notification of our team by the maternal care providers.

The primary outcomes, determined at 6 months postnatal, are 1) the difference in the level of depressive symptoms as assessed with the EPDS [22] between participants in the intervention and care as usual groups; and 2): level of parenting skills as measured by the total and subscale scores on the HOME-IT.

Assessments of secondary outcomes, to be conducted at 3 or 6 months (see Table 1), consist of the following: depression remission rates (EPDS score < 6); level of disability as assessed using the WHO Disability Assessment Scale [23]; maternal attitude and adjustment to pregnancy and motherhood as measured with the Maternal Adjustment and Maternal Attitude scale (MAMAS) [24]; quality of life using the short form of the WHO Quality of Life scale, WHOQoL-BREF; and the extent of mother–infant interactions assessed using the Postnatal Bonding Questionnaire (PBQ) [18]. The family planning and new pregnancy questionnaire (designed by the investigators) will assess what measures, if any, the adolescents have taken to prevent getting pregnant soon after the index childbirth. The Perinatal Infant Care Social Support scale (PICSS) [19] will rate the availability of social support for the adolescents as they navigate the challenge of motherhood. Assessments of the infants will include weight, height, and head circumference at birth and at 3 and 6 months of age, nutrition (history of breastfeeding), vaccinations received, and the child’s social, cognitive, and physical developmental milestones.

Study instruments have been culturally adapted using standard procedures [25, 26], including translation (into the study language, Yoruba) and back-translation of the items by a panel of bilingual experts. Even though one of the primary outcome measures, the EPDS, has been used extensively by us and others in this setting [27], we conducted a test–retest reliability of the tool among adolescent mothers. The tool was administered to 25 respondents attending for antenatal care by one set of five interviews and was re-administered to the same respondents in their homes by another set of home assessors 2–3 days after (intraclass correlation: 0.50 (95% confidence interval: −0.124 to 0.777); *p* < 0.05).

Also, with regard to the assessment of parenting skills, an inter-rater reliability exercise on the HOME-IT was conducted following training and found to be good to excellent for all of the domains. Among the five outcome assessors, independently rating 18 video-recorded HOME-IT assessments, the intra-class reliability coefficients for the domains of the tool were: Responsivity 0.89; Acceptance 0.64; Organization 0.94; Learning Materials 0.98; Involvement 0.90; Variety 0.95; and for the Total score 0.95.

### Blinding and protection against sources of bias

The following steps are being undertaken to reduce the risk of bias in this trial. First, allocation was performed by a statistician with no other involvement in the study. Second, it is recognized that allocation concealment and selection bias can be a problem in cluster randomized trials when not all participants are consented and recruited prior to the allocation of clusters. Consecutive attendees at the randomized primary maternal care clinic (MCC) are being invited to participate. Study invitees are not informed of their allocation until after establishing eligibility and providing consent to participate. Consent rates in terms of both proportion and disease severity of eligible patients in both arms of the trial are being monitored. Third, the study is designed to ensure that the risk of contamination between arms is low as patients are unlikely to move from a control to an intervention clinic due to their geographical spread and because there has been no publicity regarding the availability of the intervention in other clinics. Fourth, even though blinding of participants is clearly not possible given the nature of the intervention, however, outcome assessors are not involved in delivering the intervention and are rotated between trial participants to collect data. Fifth, our primary outcomes are the severity of depression symptoms and level of parenting skills at 6 months postnatal. We are collecting the outcome data from every participant not known to have died at the time of follow-up and who has not withdrawn consent or otherwise become unavailable, regardless of compliance with allocated treatment. Sixth, we anticipate some non-collection of primary outcome data, and while the primary intention-to-treat analyses will be without imputation of missing data, sensitivity analyses will investigate various assumptions about the missing data.

### Data collection and quality control

Data collection and capture are regulated by specific steps described in a Data Management Protocol designed to ensure data integrity and quality. Quality control of field work is implemented by research supervisors, and this includes random checks on the quality of interviews (conducted by physically observing at least 10% of the interviews conducted by a research assistant). Supervisors also work with the Data Manager to check that research assistants have correctly captured the study data.

Individual data are gathered and stored electronically. Participants consenting into the trials are informed of this and receive an ID when entering the study. Questionnaire data are collected using tablets that have been programmed to capture information directly from respondents. This will ensure accuracy and security of data collection.

### Data protection

All data are being kept anonymously, using codes to identify individuals. Data are downloaded from the tablets to a server located in the central office where they will be cleaned and stored. These datasets do not contain the allocation status of the participants, which is being kept as a separate file and only for the trial statistician. Access to the datasets is possible for members of the research team through password-protected entry.

#### Process evaluation

We are conducting a comprehensive process evaluation to assess the barriers and facilitators of scaling up the intervention. The component of the project is informed by the CFIR [28], a state-of-the-art approach for exploring implementation issues. Guided by this framework, we will explore factors such as: intervention characteristics and the process of delivering the intervention (e.g. how engaging the intervention components are to providers and users); outer setting (e.g. current standard of care, workload); inner setting (e.g. organizational structure of the facilities and acceptability of the new intervention approach to both providers and patients); characteristics of key individuals (e.g. provider attitude, patients’ compliance and adherence to treatment, etc.); and process of service delivery (e.g. fidelity to guideline specifications, supervision frequency and content, etc.). We will explore these factors with providers (*N* = 15), adolescents who recover from depression (*N* = 10), and those who do not recover (*N* = 10) 6 months after childbirth.

Monitoring of adherence to trial protocol specifications is being conducted by the Trial Manager. This follows a structured format consisting of qualitative interviews and quantitative assessments. Detailed assessment of fidelity in the delivery of the intervention specifications is being conducted. This involves the live observation and rating of 20 randomly selected therapy sessions by different providers. For this purpose, we are using the 18-item Enhancing Assessment of Common Therapeutic factors (ENACT) tool [29] to evaluate the extent to which providers are applying the skills acquired to conduct focused psychological assessment of and intervention for the adolescents. ENACT is a tool that has been developed to provide reliable and valid assessment of therapist competence in a variety of cultural and service settings. This is being done by research supervisors, appropriately trained for the purpose, sitting in during clinical encounters between maternal care providers and the depressed pregnant adolescents.

### Sample size determination for the RCT

The primary outcome considered in calculating the sample size is the level of depressive symptoms as assessed with the EPDS total score at 6 months postpartum. Based on the results of our recently concluded cluster randomized controlled trial of interventions for perinatal depression in primary care in Nigeria (EXPONATE) [15] showing a standard deviation (SD) for the EPDS score of 4.5, we estimate that a mean difference of 2.0 in the EPDS score between the two arms at 6-month postnatal follow-up will represent a clinically significant difference in depression symptoms, giving a target effect size of 0.44. About 6 months into recruitment to the current trial, there was an imbalance in the ratio of participants recruited to the trial of about 1.5 in favor of the intervention arm. An uninflated sample size of 102 in the intervention arm and 68 in the control arm will be required to provide a power of 80% at an α level of 0.05. Based on prior experience, we expect to recruit seven adolescent participants per cluster over 18 months. To take account of the cluster design, we inflate the estimated cluster size by 1 + [(*k* – 1) × ICC)], where *k* is the cluster size for analysis and ICC is the intracluster correlation coefficient. In previous use of the EPDS, we obtained an estimate of 0.03 for the ICC. Using the resulting design effect of 1.18, the estimated inflated sample size is 200 (170 × 1.18). Taking account of a projected attrition not exceeding 15% at 6 months postnatal and a resulting sample size of 230, we plan to recruit from 30 clusters.

### Data analysis

#### Qualitative analysis

To explore factors relating to the barriers and facilitators at various stages of project execution, qualitative interviews will be transcribed. Interviews will be conducted in Yoruba and will be translated into English, following which back-translation checks will be applied. The data generated will be analyzed using thematic analysis with the assistance of a qualitative software package, MAXQDA. The exploration of the findings will utilize the realist approach to address the question of “what works for whom and in what circumstance” [30, 31].

#### Quantitative analysis

The analysis and presentation of the trial will be in accordance with CONSORT guidelines [32], with the primary comparative analyses being conducted on an intention-to-treat basis and due emphasis placed on confidence intervals for the between-arm comparisons. A full analysis plan will be developed prior to completion of data collection and discussed and agreed with the TSC.

We will use descriptive statistics to assess the balance between the trial arms at baseline for both MCC and individual participant characteristics. In order to take appropriate account of the hierarchical nature of the data, we will use multivariate mixed-effects regression models (logistic or linear dependent on outcome type) to estimate recovery from depression at 6 months for the intervention group versus the control arm, adjusting for baseline depression and randomization variables. In a secondary analysis, we will further adjust for any variables that were imbalanced between trial arms at baseline. We will analyze continuous outcomes (EPDS and GAD-7) over the 6-month postnatal follow-up period using repeated-measures analysis by including follow-up occasion as a random effect in the regression model. The primary outcome will first be analyzed by intention to treat without imputation. However, we will conduct sensitivity analyses to assess the potential effect of missing data using multiple imputation methods. No interim analysis is planned except when required to explore any undesired effect that may arise during the course of the trial and such analysis is deemed necessary and approved by the TSC.

We will use descriptive statistics to explore provider competence, as assessed by ENACT. We will evaluate the provider determinants of competence by determining the demographic predictors of scores on ENACT using regression analysis.

## Discussion

There is a need to conduct research on the mental and reproductive health of adolescents, especially in low- and middle-income such as Nigeria where adolescents often constitute up to 20% of the population. Empirical data to guide national planning for adolescent health are limited in Nigeria, and more so for adolescent mental health. Furthermore, even though up to 11% of all births worldwide are to adolescents, and studies have demonstrated that they bear a large burden of common perinatal mental disorders, there is a paucity of studies demonstrating the effectiveness of interventions for such disorders as well as for improving parenting skills among adolescent mothers. RAPID is designed to fill this gap in knowledge.

## Trial status

Enrollment into the trial commenced on 15 May 2018 and is projected to end 15 November 2019. The last 6-month postnatal outcome assessment is expected to occur in August 2020.

Protocol version 3.1, 1 September 2019.

## Data Availability

The datasets used and/or analyzed during the current study are available from the corresponding author on reasonable request.

## Abbreviations

CFIR: Consolidated Framework for Implementation Research
CHEW: Community health extension workers
CHO: Community health workers
ENACT: Enhancing Assessment of Common Therapeutic factors
EPDS: Edinburgh Postnatal Depression Scale
EXPONATE: Expanding care for perinatal women with depression
GAD-7: Generalized Anxiety Disorder Assessment scale
HOME-IT: Home Inventory for Measurement of Home Environment, Infant–Toddler version
LGA: Local government area
LMIC: Low and middle-income country
MCC: Maternal care clinic
mhGAP-IG: World Health Organization Mental Health Gap Action Programme, intervention guide
PBQ: Postnatal Bonding Questionnaire
PHCW: Primary health care workers
PICSS: Parental Infant Care Social Support Scale
RAPID: Responding to the challenge of Adolescent Perinatal Depression
TMC: Trial Management Committee
TSC: Trial Steering Committee
WHOQoL-BREF: World Health Organization Quality of Life-BREF
